# Supplementary material for: Unlocking the Potential of Kinase Targets in Cancer: Insights from CancerOmicsNet, an AI-Driven Approach to Drug Response Prediction in Cancer
Source: Cancers (Basel). 2023 Aug 10;15(16):4050. doi: 10.3390/cancers15164050 (PMC10452340; doi:10.3390/cancers15164050)
Supplement: Supplementary file 1 [file cancers-15-04050-s001.zip › Supplementary Figures S1-S3.pdf]

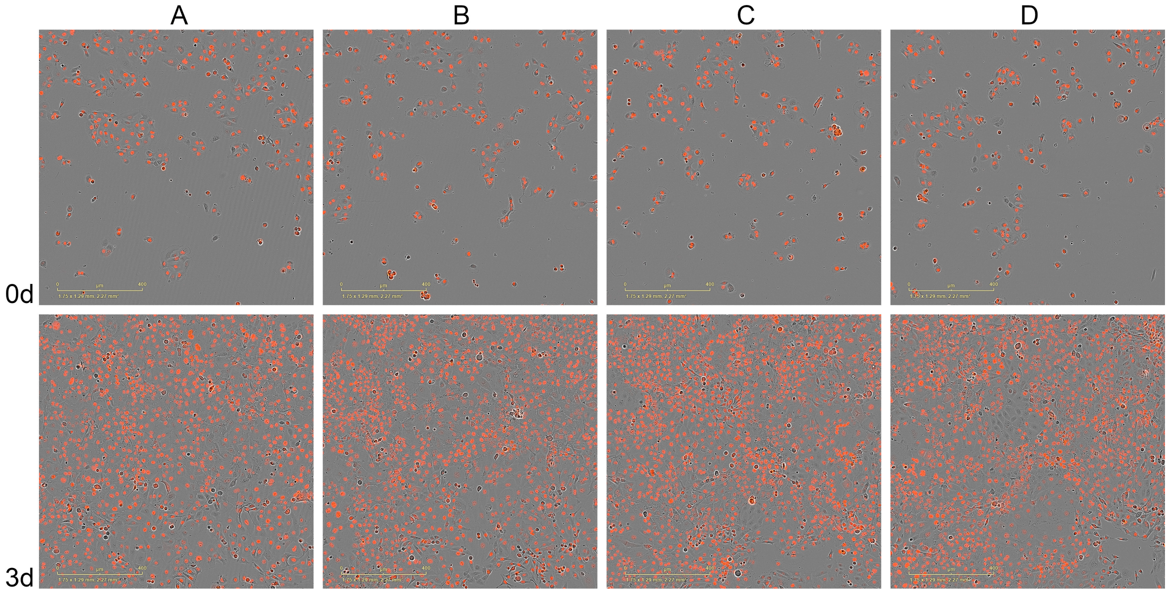

Figure S1. Normalized cell counts for (A) HCC70 treated with PI-103, (B) Panc 04.03 treated with PP1, and (C) DU 145 treated with XMD8-9. Drug treatment at 10 μM, 3.162 μM, and 1 μM is compared to the vehicle treatment (DMSO). Gray bars represent cell counts recorded before the treatment (0d) while white bars show counts 3 days after the treatment (3d).

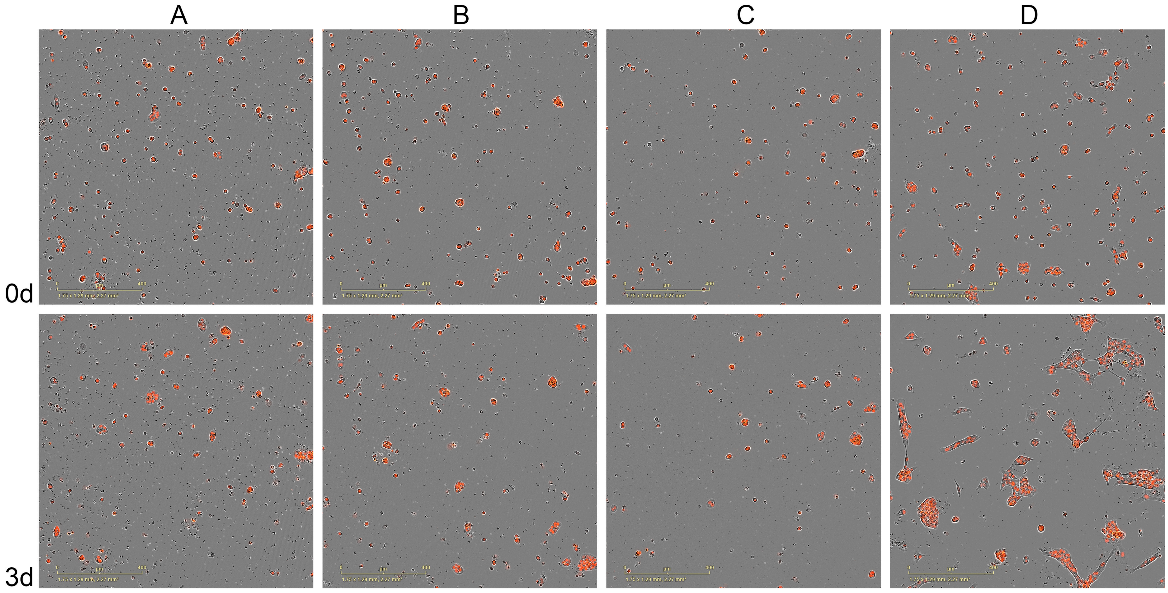

Figure S2. Microscopy images of HCC70 cells treated with PI-103. Drug treatment at (A) 10 μM, (B) 3.162 μM, and (C) 1 μM is compared to the vehicle treatment (D). The first row shows images recorded before the treatment and the second row shows images taken 3 days after the treatment.

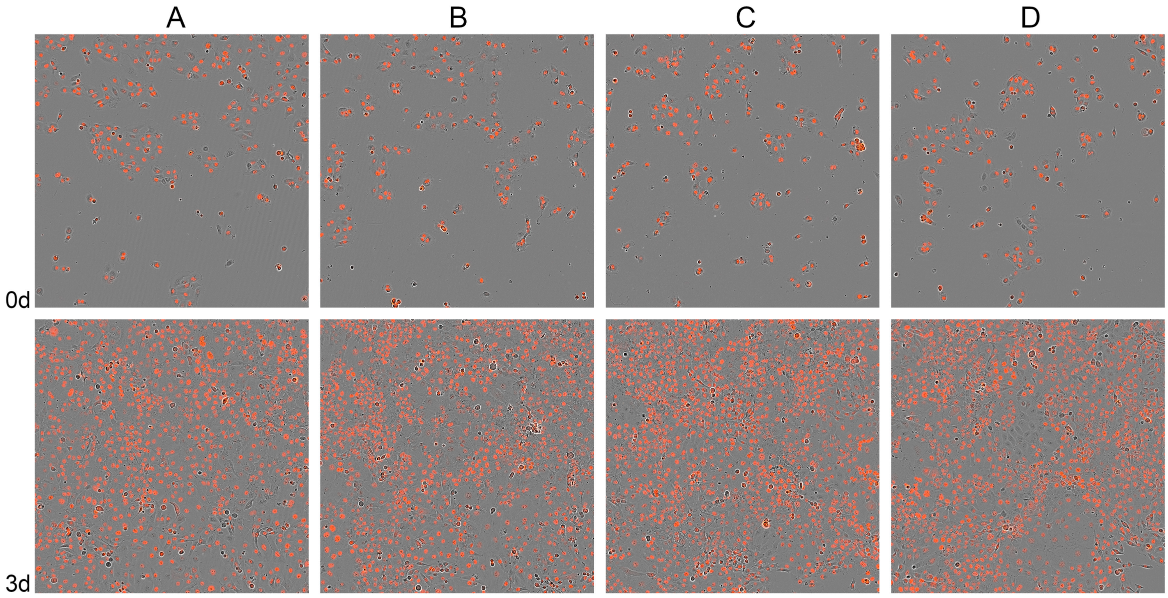

Figure S3. Microscopy images of DU 145 cells treated with XMD8-92. Drug treatment at (A) 10 μM, (B) 3.162 μM, and (C) 1 μM is compared to the vehicle treatment (D). The first row shows images recorded before the treatment and the second row shows images taken 3 days after the treatment.
