# Supplementary material for: Unlocking the Potential of Kinase Targets in Cancer: Insights from CancerOmicsNet, an AI-Driven Approach to Drug Response Prediction in Cancer
Source: Cancers (Basel). 2023 Aug 10;15(16):4050. doi: 10.3390/cancers15164050 (PMC10452340; doi:10.3390/cancers15164050)
Supplement: Supplementary file 1 [file cancers-15-04050-s001.zip › SupplementaryMethods.pdf]

Supplementary Methods

Cell lines and culture conditions. Cultured cell lines were maintained under standard laboratory conditions: 37 °C temperature and 5% CO_2 in a water jacketed tissue culture incubator. Pan 04.03 cells (ATCC, CRL-2555) were cultured in RPMI-1640 (ATCC, 30-2001) supplemented with 20 units/mL of human recombinant insulin and 15% fetal bovine serum. HCC70 cells (ATCC, CRL-2315) were cultured in RPMI-1640 (ATCC, 30-2001) supplemented with 10% fetal bovine serum. DU 145 cells (ATCC, HTB-81) were cultured in Eagle's minimum essential medium (EMEM) (ATCC, 30-2003) supplemented with 10% fetal bovine serum.

Lentivirus transduction. The IncuCyte nuclight red lentivirus reagent (Sartorius, Catalogue No. 4476) was obtained and used to transduce Pan 04.03, DU 145, and HCC70 cell lines at a multiplicity of infection (MOI) of 1. Briefly, 3×10^5 cells were seeded into a well of a 6-well plate (Corning, Catalogue No. 353046). After overnight incubation, 200 µL of lentivirus particles consisting of approximately 3×10^5 transducing units were added to the cells and incubated overnight. The next day, the media was replaced, and cells were allowed to expand for 3 days. Subsequently, cells were selected for transduction by adding 1 µg/mL puromycin. Nuclear fluorescence was observed using an inverted fluorescent microscope to track transduction efficiency and cells were continually selected for further analysis.

Drugs. Kinase inhibitors were purchased from suppliers: PP1 (MedChemExpress, HY-13804), XMD8-93 (MedChemExpress, HY-14443), and PI-103 (MedChemExpress, HY-10115). The drugs were obtained as stock solutions of 10 mM in DMSO.

Live cell imaging and cell counting. Cells were seeded at densities of 312, 156, and 78 cells/well in 384-well plates (Corning, Catalogue No. 3764) with duplicate wells containing 20 µL of media and incubated overnight. The next day, a 2× dilution series of the drugs was added to the cells, resulting in final concentrations of 1 µM, 3.162 µM, and 10 µM. Cells were then imaged for 72 hours using the IncuCyte S3 system with a 400 ms acquisition time in the red channel and a 10× objective. Adherent cell-by-cell analysis was performed to quantify the number of red nuclei in each well throughout the 72-hour observation period. Cell-by-cell analysis software is provided as part of the IncuCyte system.
